# Cost-effectiveness of CRAG-LFA screening for cryptococcal meningitis among people living with HIV in Uganda

**DOI:** 10.1186/s12879-017-2325-9

**Published:** 2017-03-23

**Authors:** Anu Ramachandran, Yukari Manabe, Radha Rajasingham, Maunank Shah

**Affiliations:** 1 Johns Hopkins University School of Medicine, 725 N. Wolfe St. PCTB building-224, Baltimore, MD 21205 USA; 2 Infectious Diseases Institute, Makerere University College of Health Sciences, Kampala, Uganda; 3 Division of Infectious Diseases and International Medicine, Department of Medicine, University of Minnesota, Minneapolis, MN USA

## Abstract

**Background:**

Cryptococcal meningitis (CM) constitutes a significant source of mortality in resource-limited regions. Cryptococcal antigen (CRAG) can be detected in the blood before onset of meningitis. We sought to determine the cost-effectiveness of implementing CRAG screening using the recently developed CRAG lateral flow assay in Uganda compared to current practice without screening.

**Methods:**

A decision-analytic model was constructed to compare two strategies for cryptococcal prevention among people living with HIV with CD4 < 100 in Uganda: No cryptococcal screening vs. CRAG screening with WHO-recommended preemptive treatment for CRAG-positive patients. The model was constructed to reflect primary HIV clinics in Uganda, with a cohort of HIV-infected patients with CD4 < 100 cells/uL. Primary outcomes were expected costs, DALYs, and incremental cost-effectiveness ratios (ICERs). We evaluated varying levels of programmatic implementation in secondary analysis.

**Results:**

CRAG screening was considered highly cost-effective and was associated with an ICER of $6.14 per DALY averted compared to no screening (95% uncertainty range: $-20.32 to $36.47). Overall, implementation of CRAG screening was projected to cost $1.52 more per person, and was projected to result in a 40% relative reduction in cryptococcal-associated mortality. In probabilistic sensitivity analysis, CRAG screening was cost-effective in 100% of scenarios and cost saving (ie cheaper and more effective than no screening) in 30% of scenarios. Secondary analysis projected a total cost of $651,454 for 100% implementation of screening nationally, while averting 1228 deaths compared to no screening.

**Conclusion:**

CRAG screening for PLWH with low CD4 represents excellent value for money with the potential to prevent cryptococcal morbidity and mortality in Uganda.

## Background

Cryptococcal meningitis (CM) constitutes a significant source of global morbidity and mortality for people living with HIV (PLWH), particularly in resource limited settings [1]. Nearly 1 million cases of cryptococcal disease with 625,000 deaths are estimated to occur annually, surpassing tuberculosis and other opportunistic infections in some highly prevalent areas [1]. Of these, the vast majority of cases occur in sub-Saharan Africa, where CM is the cause of 10–44% of HIV-associated deaths [1–4]. New cost-effective approaches for prevention, detection and treatment of cryptococcal disease are needed.

Several factors contribute to high cryptococcal morbidity and mortality, including difficulty with accurate diagnosis and complicated disease management consisting of hospitalization, serial lumbar punctures, and expensive treatments that are difficult to access such as amphotericin and flucytosine, both with significant side effects [5, 6]. CM mortality remains high in both resourced (9–38%) and resource-limited settings (30–59%) despite increasing access to ART, likely due to late presentation [7, 8]. Universal primary prophylaxis (UPP) with fluconazole has not demonstrated a clear mortality reduction and incurs significant costs and side effects [7, 9, 10]. Strategies that allow targeted early interventions to identify those at greatest risk for cryptococcal disease are thus warranted.

To this end, cryptococcal antigen (CRAG) screening of immunosuppressed HIV-infected (CD4 < 100 cells/μL) allows early identification of individuals with cryptococcal infection or those at risk for increased mortality. In 2011, the cryptococcal antigen Lateral Flow Assay (IMMY CrAg LFA, Norman, OK, 2011), was developed. This is a rapid dipstick test, approved by the Food and Drug Administration, with a very high sensitivity and specificity for detecting cryptococcal glucuronylmannan in serum [11–16]. CRAG LFA is heat-stable, inexpensive (estimated $2.50 per test), and requires minimal training for optimal use in resource-limited settings. Several studies have demonstrated its effectiveness in detecting cryptococcal antigenemia and diagnosing cryptococcal disease [17, 18].

CRAG is detectable in the blood 3 weeks before onset of meningitis, and is an independent predictor of meningitis and death [19–23]. CRAG screening targeting those HIV-infected with a CD4 count <100 cells/uL is now recommended by the WHO, and several national HIV guidelines [24]. For those who are CRAG positive, preemptive treatment with fluconazole (800 mg for 2 weeks, followed by 400 mg for 8 weeks) reduces mortality [25].

Despite the WHO recommendations, implementation and scale-up of CRAG screening remains limited in Sub-Saharan Africa. Fewer than 30% of eligible patients receive screening, partly due to concerns regarding cost and feasibility of incorporation into routine HIV care [26]. Previous economic evaluations from middle-income settings have suggested that incorporating CRAG-LFA screening into HIV care may be cost-effective at current willingness to pay thresholds [27]. There is a lack of data from low-income settings in Sub-Saharan Africa where disease burden is highest [28–30]. Meya et al. previously assessed costs of CRAG screening using a latex agglutination assay ($16 per test) in Uganda as part of a cohort study; however, the cost-effectiveness of population-level implementation was not evaluated [28]. The impact of preemptive treatment, relapse, or loss to follow-up on the cost-effectiveness of CRAG screening has not been modeled, and an economic analysis of the impact and cost of scaling up current screening protocols is important for guiding policy decisions. Furthermore, alternative implementation algorithms including intensified diagnostic evaluation of all CRAG-positive patients or full CM treatment for all CRAG-positive patients may improve morbidity and mortality for patients with sub-clinical CM disease. Current recommendations do not have provisions for such intensified diagnostics, and have not evaluated their cost-effectiveness.

We thus sought to evaluate the costs and cost-effectiveness of implementing CRAG screening for PLWH with advanced immunosuppression (including preemptive treatment for those CRAG positive) compared to current practice in Uganda. Additionally, we sought to explore the health-system costs of CRAG screening at various levels of programmatic implementation as well as alternative algorithms for screening.

## Methods

**Fig. 1:**
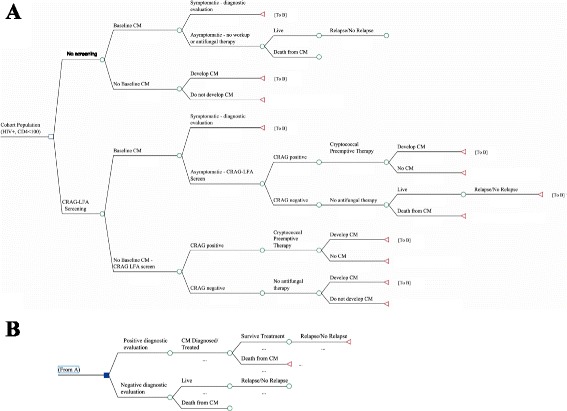
Model Schematic. Abbreviations: CRAG-LFA—cryptococcal antigen lateral flow assay, CM—cryptococcal meningitis, ART—antiretroviral therapy. Decision-analytic model schematic. We modeled progression or relapse of CM over a 5 year time period for a cohort of PLWH with CD4 < 100. In both model arms, symptomatic patients at baseline receive evaluation for CM assumed to include a lumbar puncture (LP), and treatment if diagnosed with CM. We assumed ART initiation in all arms. The model compares two interventions for prevention of cryptococcal morbidity for those without a baseline diagnosis of CM: 1) No screening, in which patients receive no CM screening or prophylaxis 2) CRAG-LFA, in which all patients receive serum CRAG-LFA screening. Individuals with positive CRAG were assumed to receive the cryptococcal preemptive treatment for cryptococcal antigenemia with fluconazole 800 mg for 2 weeks, followed by fluconazole 400 mg for 8 weeks. CRAG-negative individuals receive no further antifungal therapy. **a** outlines the two main model arms. **b** demonstrates the diagnostic evaluation and treatment for suspected CM cases

We conducted a cost-effectiveness analysis from a health-systems perspective using a decision-analytic model (Fig. 1) with a target population of HIV-infected, ART-naïve Ugandan patients with a baseline CD4 < 100 cells/μL. We used a 1-year time-frame for the intervention, and evaluated outcomes from cryptococcal disease over a 5-year period. Disability-adjusted life years (DALYs) were analyzed across a lifetime horizon of the cohort representing PLWH on ART. The model was developed and analyzed using TreeAge software (TreeAge Software Inc., Williamstown, MA, USA).

### Decision-Tree Model

Two practices for PLWH with CD4 < 100 cells/uL were compared: No screening vs universal CRAG screening followed by preemptive treatment for those CRAG positive.

In both model arms, patients were stratified by prevalence of baseline CM disease and cryptococcal antigenemia, based on currently available data from published literature [20, 25, 28, 29] (Table 1). All patients in both model arms were initiated on ART per current guidelines in Uganda. Regardless of CRAG screening strategy, all patients with symptoms concerning for CM at presentation (eg. headache, fever, or neck stiffness) were assumed to receive a lumbar puncture (LP) and treatment for baseline CM if diagnosed, consisting of Amphotericin B 0.7 mg/kg/day for 2 weeks, followed by Fluconazole 800 mg for 3 weeks and Fluconazole 400 for 9 weeks, with potential for future relapse [30]. All patients without initial CM disease could progress to develop CM, based on current literature estimates and adjusted based on model interventions such as receiving preemptive treatment. Given uncertainty in long-term progression rates, we focused on short/medium term progression over 5 years [31]. Patients that progressed to develop CM could either be detected and treated, or lost to follow-up. Untreated CM among PLWH was assumed to have 100% mortality [8]. Within each arm of the model, PLWH that were successfully treated for CM disease could still go on to have disease relapse (Fig. 1).

**Table 1:** Key Parameter Estimates

Parameter name	Value	Range	Source
Epidemiology			
Number of patients enrolling in HIV care in Uganda per year	245,600	150,000–350,000	[34]
Total number of patients enrolling in HIV care per year with CD4 < 100 (% of total)	61,400 (25%)	13,000 (5%) -105,000 (30%)	[34]
Average age of cohort	37	32–44	[41]
Life Expectancy in years^a^ (on ART)	19.1	17–23	[41]
CRAG-positive prevalence (at baseline)	8.80%	1–20%	[20, 29, 31, 32]
Percentage of CRAG+ patients with baseline CM disease	25%	0–50%	[33, 45]
Proportion developing CM among CRAG positive patients without treatment^b^	34%	10–75%	[21, 23, 31, 29]
Relative reduction in CM development among CRAG positive patients on CPET^b,c^	65%	10–90%	[51, 26, 29, 31]
Proportion developing CM among CRAG negative patients^b^	0.8%	0–2%	[10, 21, 29]
Treatment			
Survival of diagnosed CM with full CM treatment	70%	50–90%	[30]
Survival of CM without treatment	0%		
Relapse rate for treated CM^d^	12.50%	9–16%	[26, 43]
Disability Weights			
CM disease	0.615	0.46–0.77	[52, 53]
HIV (on ART)	0.053	0.039–0.066	[52, 53]
CM treatment	0.05	0.0375–0.0625	[52, 53] (Assumption)
Costs			
CRAG-LFA	$2.52	$1.50–$10.00	[38]
Lumbar Puncture	$8.08	$6.03–$10.42	[35, 38]
CM Diagnosis (lab and staff costs)	$7.07	$2.42–$12.79	[35–38]
Preemptive treatment: Fluconazole 800 mg daily for 2 weeks, then 400 mg daily for 8 weeks	$22.22	$17.00–$33.00	[24, 35]
CM Treatment: Amphotericin B 0.7 mg/kg/day for 2 weeks, then fluconazole 800 mg for 3 weeks and fluconazole 400 for 9 weeks	$343.28	$200–$600	[33–35]

*Abbreviations*: *CRAG-LFA* cryptococcal antigen lateral flow assay, *CPET* WHO Cryptococcal Pre-emptive therapy, *CM* cryptococcal meningitis, *ART* antiretroviral therapy

^a^Life expectancy was estimated to be reduced by 25% in CRAG positive individuals, independent of development of CM, to account for higher observed mortality in this population in recent cohort studies [20]

^b^CRAG-LFA testing was a component of only the CRAG-LFA screening intervention; However, risk of progression to CM for all model arms was stratified based on epidemiologic data on prevalence of baseline CRAG positivity

^c^Absolute and relative risk reduction were calculated based on available studies of fluconazole therapy in CRAG positive patients [26, 29, 31]. An estimated 15% of patients in all model arms were considered lost to follow up over the time horizon of the model, with higher rates of CM development and relapse. Sensitivity analysis was conducted around these parameters

^d^Assuming a 75% case detection rate for symptomatic CM disease

In order to evaluate the impact and cost-effectiveness of CRAG screening, rates of diagnosis and/or development and treatment of CM differed between the model arms based on usage of CRAG screening. In the ‘no screening’ arm, no CRAG screening was performed and no prophylactic antifungal therapy was administered. Asymptomatic patients were assumed to receive no further immediate cryptococcal related care, while symptomatic patients received evaluation as described above; patients without baseline CM were considered at risk for development of CM with rates dependent on prevalence of (undetected) cryptococcal antigenemia.

In the CRAG screening intervention arm, patients received a serum CRAG test using the LFA to identify cryptococcal antigenemia. In the base case, symptomatic CRAG-positive patients received further evaluation as described above, while asymptomatic CRAG-positive patients received no further evaluation. All asymptomatic CRAG-positive patients received preemptive treatment for cryptococcal antigenemia (fluconazole 800 mg for 2 weeks followed by 400 mg for 8 weeks) along with ART initiation per current Ugandan guidelines [24, 30]. We incorporated the protective effect of preemptive treatment as a reduction in the risk of developing CM, and as partial treatment for those with subclinical baseline CM [25, 26, 29–33]. CRAG negative patients began ART without further testing or treatment, and were at risk for development of CM based on literature estimates (Table 1).

To aid in programmatic implementation, we conducted secondary analysis to explore alternative interventions for individuals with baseline CRAG positivity in the CRAG-screening intervention arm, including an intensified case-finding algorithm consisting of an LP for evaluation of CM in all CRAG positive patients regardless of symptoms (in base case analysis, only symptomatic patients receive LP and asymptomatic patients receive preemptive therapy). In this algorithm, CRAG positive patients diagnosed with CM disease after LP receive full CM treatment, while CRAG positive patients without a diagnosis of CM after LP are treated with preemptive therapy. We additionally explored provisions for full CM treatment to all CRAG positive patients, without requiring LP or additional diagnostic workup. The effect of each of these clinical algorithms on ICER values for CRAG-LFA screening was determined. Policy makers must also consider the costs and impact of scaling up new screening strategies. As such, in additional secondary analysis, we also modeled the net costs, effects, and cost-effectiveness of population level scale-up of CRAG screening, by varying the percentage of individuals (accessing HIV care in a given year) receiving CRAG screening, and compared these to the current practice arm. The absolute number of Ugandans enrolling in HIV care per year with CD4 < 100 was estimated to be 61,400 and was varied widely in sensitivity analysis [34].

### Epidemiologic, diagnostic, and treatment parameters

Key model parameters, values, and ranges are shown in Table 1. Variables including prevalence of cryptococcal antigenemia, percentage uptake, and percent of HIV patients with CD4 count <100 cells/μL were varied widely in sensitivity analysis to incorporate findings from multiple countries. For variables with limited data, uncertainty ranges were determined by varying base case values by ±75%. Probabilistic sensitivity analysis using Monte-Carlo simulation was conducted to simultaneously vary all parameters across their ranges to generate 95% uncertainty ranges (UR) for outcome estimates of costs, DALYs and cost-effectiveness.

### Costs

Costs were derived from literature estimates as well as invoices from the Infectious Disease Institute in Kampala, Uganda and are presented in 2016 US dollars. Costs of the CRAG LFA, lumbar puncture, and CM diagnosis were based on estimates accounting for personnel, materials, and outpatient visit costs, and are summarized in Table 1. Medication costs were based on prices available in Uganda from the Joint Medical Service [35–39]. Full CM treatment cost included the price of medication (Amphotericin B and Fluconazole), hospitalization, and therapeutic laboratory monitoring. Lifetime costs of ART and HIV care were not included in the base case analysis. However, to determine the impact of ART costs on the cost-effectiveness of CRAG screening, secondary analyses were performed to calculate the ICER inclusive of lifetime ART cost. Costs for future years were discounted at 3% [40–42].

### Outcomes

The primary outcomes of the model were DALYs calculated based on disability attributable to CM disease with associated treatments, and years of life lost from CM-associated mortality (Table 1). We assumed near full life expectancy for individuals without CM disease with ART, based on site-specific data in Uganda for HIV-infected persons [41].

Cost-effectiveness was represented by the incremental cost-effectiveness ratio (ICER) comparing model arms, and assessed relative to WHO’s suggested country-specific willingness-to-pay (WTP) threshold, defined in our analysis as per-capita Ugandan gross domestic product (GDP $572) per DALY averted [43, 44].

## Results

### Costs

Projected costs are summarized in Table 2. The incremental health system cost of the CRAG screening intervention in the base case scenario (i.e.100% implementation of CRAG screening for 1 year; total costs inclusive of a 5 year time period of downstream costs) was projected to be $1.52 (95%UR: $-5.46 to $9.42) more than ‘no screening’ per patient,. The diagnostic costs of the CRAG-LFA testing algorithm ($2.71 per person, inclusive of all diagnostic workup costs) were largely offset by averted costs associated with reduced development and treatment of CM when compared to costs of no screening (treatment costs for CRAG-LFA arm: $7.90 per person, compared to $8.85 for no screening).

**Table 2:** Costs, Effects, and ICER values of implementing universal CRAG screening

Intervention	Total Cost (95% UR)^a,c^	Incremental Cost^c^	DALYs	Incremental Effectiveness (DALYs averted)	ICER (cost per DALY averted)^c^
No screening	9.24 (7.31to 18.40)	REFERENCE	8.55 (6.70 to 10.90)	REFERENCE	REFERENCE
CRAG screening^b^	10.76 (8.39 to 12.29)	1.52 (−5.46 to 9.42)	8.30 (6.41to 10.79)	25 DALYs averted per 100 participants (13 to 47)	6.14 (−20.32 to 36.47)

*Abbreviations*: *CP* current practice, *CRAG-LFA* cryptococcal antigen lateral flow assay, *DALY* disability adjusted life-year, *ICER* incremental cost-effectiveness ratio

^a^Total costs represent total health systems costs, inclusive of diagnostic testing and treatment costs related to diagnosed cryptococcal antigenemia and/or cryptococcal meningitis over a 5 year time period, but excludes lifetime ART costs. DALYs were evaluated over a lifelong time horizon. Future years are discounted by 3% and ART costs are not included in base case analysis

^b^The CRAG-LFA intervention consists of screening all cohort patients for cryptococcal antigenemia with CRAG-LFA, followed by cryptococcal pre-emptive therapy (CPET) for those who screen positive

^c^In secondary analysis, the total costs inclusive of lifetime ART costs were $5772 and $5991 (incremental of139.48) for CP and CRAG-LFA screening arms, respectively. The ICER inclusive of lifetime ART costs was 558 per DALY averted

### Effectiveness

CRAG screening, implemented for all eligible individuals, resulted in improved health benefits compared to no screening (25 DALYs averted per hundred individuals), as summarized in Table 2. In the CRAG screening intervention arm, use of preemptive therapy among CRAG positive patients was projected to reduce CM development and thus early CM mortality. We estimated that implementation of CRAG screening resulted in a relative risk reduction in cryptococcal-associated mortality of 44% compared to CP (Absolute CM mortality projections: No screening 45 deaths per 1000 PLWH, CRAG screening 25 deaths per 1000 PLWH).

### Cost-effectiveness

In the base case analysis, 100% implementation of CRAG screening (with preemptive therapy for CRAG-positive patients) was highly cost-effective compared to No screening ($6.14 per DALY averted, 95% UR: -$20.32 to $36.47) at current WTP thresholds for Uganda (Table 2). In secondary analysis, when including cost of lifetime ART, CRAG screening cost approximately $557 per DALY averted, still below the WTP threshold for cost-effectiveness in Uganda.

### Secondary analysis

In the base case we assumed all asymptomatic CRAG-positive patients received preemptive treatment in the CRAG screening arm, without further evaluation for subclinical baseline CM. We explored alternative implementation algorithms for intensified diagnosis and treatment for CRAG-positive patients. If all CRAG-positive patients received a LP for intensified diagnosis of CM (with full CM treatment if diagnosed), the ICER for CRAG screening rose to $32 per DALY averted compared to No screening. Alternatively, if all CRAG positive patients were treated for CM (i.e. Amphotericin followed by fluconazole) irrespective of any additional evaluation, the ICER rises to $75 per DALY averted compared to No screening.

In further secondary analysis, we calculated the costs and effects of CRAG screening at four levels of programmatic scale-up (25, 50, 75, and 100% of eligible individuals, Table 3). We found that the incremental costs and DALYs averted were affected proportionally by percentage uptake of CRAG screening, and consequently the ICER for each level of implementation remained unchanged at $6.14 per DALY averted. However, the absolute costs and impact of varied by different levels of population level coverage of CRAG screening. The total (discounted) health system cost for an annual estimate of persons with HIV and CD4 < 100 cells/uL enrolling into care (inclusive of screening, further diagnostic evaluation when indicated, and treatment costs) of CRAG screening in Uganda was estimated at $651,454 at 100% uptake ($473,394, $303,930, $146,132 for 75%, 50%, 25% uptake respectively). Total mortality among this cohort from CM disease improved at each level of programmatic implementation (CM deaths averted compared to ‘no screening’: 307 at 25%, 614 at 50%, 921 at 75%, 1228 at 100% uptake). These estimates varied based on the estimated number of people enrolling in HIV care in Uganda as well as the estimated percentage with CD4 < 100 (Table 3).

**Table 3:** Absolute Costs and Effects for Various Levels of CRAG Screening

Implementation of CRAG screening (%)					Universal Screening
	0%	25%	50%	75%	100%
Number of Patients eligible for CRAG screening(CD4 < 100) enrolling in care per year	61,400 (13,000–105,000)	61,400 (13,000–105,000)	61,400 (13,000–105,000)	61,400 (13,000–105,000)	61,400 (13,000–105,000)
Number of patients receiving CRAG screening	0	15,350 (3250–26,250)	30,700 (6500–52,500)	46,050 (9750–78,750)	61,400 (13,000–105,000)
Total cost per patient^a,b^	$9.24 (7.31–18.40)	$9.52 (0.87–26.87)	$9.90 (1.28–27.29)	$10.28 (1.54–27.40)	$10.76 (1.85–28.26)
Incremental Cost per patient, compared to No screening^a,b^	REFERENCE	$0.37 (−6.44–8.47)	$0.75 (−6.03–8.89)	$1.13 (−5.77–9.09)	$1.52 (−5.46–9.42)
Total Program Cost	$559,968 (95,000–1,900,000)	$584,528 (123,000–1,930,000)	$607,860 (154,000–1,960,000)	$631,192 (178,000–1,990,000)	$651,454 (198,000–2,100,000)
Total Incremental Program Costs compared to No screening^a,b^	REFERENCE	$24,560 (12,000–36,000)	$47,892 (30,000–64,000)	$71,224 (54,000–96,000)	$91,486 (76,000–104,000)
DALYs accumulated per patient	8.55 (6.70–10.90)	8.48 (1.59–10.87)	8.42 (6.53–10.84)	8.36 (6.47–10.82)	8.3 (6.41–10.79)
Total DALYs averted compared to No screening	REFERENCE	4298	7982	11,666	15,350
Deaths from CM (proportion of cohort)	2763 (0.045)	2456 (0.04)	2149 (0.035)	1842 (0.03)	1535 (0.025)
CM deaths averted compared to No screening	REFERENCE	307	614	921	1228

*Abbreviations*: *CRAG-LFA* cryptococcal antigen lateral flow assay, *DALY* disability adjusted life-year, *ICER* incremental cost-effectiveness ratio

^a^Total costs represent total health systems costs, inclusive of diagnostic testing and treatment costs related to diagnosed cryptococcal antigenemia and/or cryptococcal meningitis over a 5 year time period, but excludes lifetime ART costs. Future years are discounted by 3% and ART costs are not included in base case analysis. Costs stratified by source: No screening – Diagnostic costs $0.27 per person, $16,500 total, Treatment costs $8.85 per person, $543,000 total. Universal CRAG screening – Diagnostic costs $2.71 per person, $166,000 total, Treatment costs $7.90 per person, $485,000 total

^b^Total cost per patient including lifetime ART: $5772 at 0%, $5807 at 25%, $5842 at 50%, $5876 at 75%, $5911 at 100%. Projected total program cost including lifetime ART: $354,400,800 at 0%, $89,133,613 at 25%, $179,318,700 at 50%, $270,601,313 at 75%, $362,935,400 at 100%

### Sensitivity analysis

**Fig. 2:**
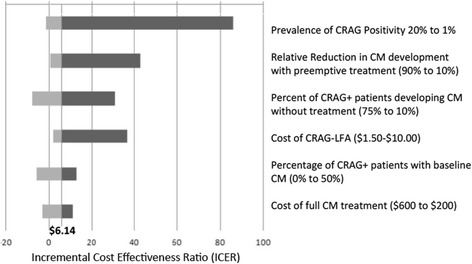
Tornado Diagram demonstrating Impact of Model Variables on ICER. Abbreviations: CRAG-LFA –cryptococcal antigen lateral flow assay, CM – cryptococcal meningitis, CRAG – cryptococcal antigen. Tornado diagram demonstrating the effect of varying six pertinent variables on the ICER value for CRAG screening compared to CP. The vertical line at $6.14 represents the ICER for the base case analysis

We explored the impact of parameter uncertainty on our base case cost-effectiveness estimates (at 100% implementation) in one-way and multi-way sensitivity analyses (see Fig. 2 for selected one-way analyses). We did not identify any scenarios in which CRAG screening would not be considered highly cost-effective (i.e. where ICER dropped below the WTP threshold).

The variables that most impacted the ICER for CRAG screening included prevalence of cryptococcal antigenemia, relative reduction in CM progression among those who received preemptive therapy, and cost of CRAG-LFA testing. However, CRAG screening remained highly cost-effective at current WTP thresholds even at a CRAG prevalence of 0.5%, CRAG LFA cost of $9.80, and only 10% efficacy of preemptive treatment. Our model projected that if the proportion of CRAG positive cohort patients developing CM over a 5-year time period (despite ART usage) rose above 44%, CRAG screening became cost-saving compared to No screening. CRAG screening also became cost-saving if preemptive therapy reduced CM development by 90% compared to no therapy.

In probabilistic sensitivity analysis, CRAG screening was considered cost-effective compared to ‘no screening’ 100% of the time at a WTP threshold of GDP per capita. CRAG screening was considered cost-saving (both less expensive and more effective than No screening) in approximately 32% of simulations.

## Discussion

Diagnosis and management of CM remains a significant clinical and infrastructural problem in Sub-Saharan Africa. Our study suggests that for HIV-infected patients with CD4 counts <100 cells/μL, incorporation of routine CRAG screening using the LFA would be highly cost-effective compared to no screening. Our model projects that CRAG screening with preemptive therapy for CRAG-positive individuals could reduce relative risk of CM mortality among PLWH with CD4 < 100 by over 40% while increasing costs by less than $2.00 per patient compared to no screening, suggesting that CRAG screening represents excellent value for money for HIV programs in these regions.

Our analysis also provides a unique assessment of alternative implementation algorithms for CRAG screening that have not been previously modeled, including intensified diagnostic evaluation with LP and providing full empiric treatment for CM among all CRAG-positive individuals. At $32 per DALY averted, incorporation of LP for all CRAG-positive patients remained well below WTP thresholds for Uganda and improved outcomes for patients with sub-clinical CM disease. While treatment of all CRAG-positive patients with full CM treatment also improved outcomes, it more than doubled the ICER compared to intensified diagnosis ($75 per DALY averted) with only a marginal mortality benefit. Our results suggest that, where feasible, intensified diagnostic evaluation of CRAG-positive patients with LP can improve detection of sub-clinical CM disease while remaining cost-effective [45].

Sensitivity analysis identified important parameters underlying the cost-effectiveness of CRAG screening. The prevalence of CRAG positivity among the cohort population had a significant influence on the ICER – as disease prevalence drops, CRAG screening identifies fewer high-risk patients and leads to fewer absolute benefits. Despite this, CRAG screening remained cost effective at the WTP threshold for Uganda even if prevalence declined to 1%; this result suggests that CRAG screening need not be limited to high-prevalence areas. Estimates of CRAG prevalence from various cohorts in Thailand and Sub-Saharan Africa including Ghana, Kenya, Ethiopia, Nigeria, and South Africa have reported a range of cryptococcal antigenemia from 2.2 to 21%, indicating that our results could be generalizable across much of Sub-Saharan Africa [7, 46]. The US and UK have previously estimated CRAG prevalences of 2.9 and 5% among cohorts of PLWH with CD4 < 100, suggesting that routine CRAG screening may be cost-effective in these populations as well, though further evaluation is warranted given the significant differences in practice and costs [47, 48].

There are several limitations to our study. Like all cost-effectiveness analyses, we are limited by the availability of data to inform estimates for key model parameters. When data were limited, we incorporated large ranges and conducted sensitivity analyses to assess their impact on cost-effectiveness. Overall, our model projections are consistent with empirically collected data from studies evaluating CRAG screening (31–57 deaths per 1000 PLWH) [2, 3, 8]. While base case analysis did not include ART costs, we performed secondary analysis to incorporate these costs into each intervention and calculate the ICER inclusive of ART cost. Our model assumes optimal implementation and uptake of CRAG screening; however this may not be possible in resource-limited settings. Additional research is needed to examine infrastructural and programmatic barriers associated with implementing a CRAG-LFA screening program.

Our study has significant strengths and differs from previous analyses in several important ways. First our model focuses on population-level effects of scaling-up CRAG screening in a low-income country in Sub-Sarahan Africa, a region with high cryptococcal burden and approximately 500,000 of the 625,000 yearly CM deaths [1]. Furthermore, we modeled the incorporation of preemptive therapy for clinical management of cryptococcal antigenemia and explored cost-effectiveness at varying degrees of preemptive treatment efficacy. We also incorporated relapse and loss-to-follow-up into our projections and modeled the intervention at various degrees of scale-up to estimate realistic implementation efforts. Our findings build on data from higher income settings and demonstrate the cost-effectiveness and projected implementation effects of CRAG-LFA screening for a high prevalence, low-income region.

Despite WHO recommendations for universal CRAG screening among eligible patients, implementation is of paramount importance and remains a challenge in overburdened, under-resourced clinics. Despite known survival benefit with CRAG screening, successful national implementation will require education of healthcare workers, training of laboratory workers, purchasing of CRAG LFA kits, and sufficient supply of fluconazole and ART. In our experience stock-outs of fluconazole are a common limiting factor. CRAG screening programs with high rates of loss to follow up, have proven unsuccessful [45]. Without an advocate in each clinic for those particularly vulnerable patients with a CD4 < 100 cells/uL, it remains challenging to successfully implement CRAG screening programs outside of research studies. Finally, CRAG screening programs need to be closely linked to ART initiation and retention in care. ART remains the most important intervention to decrease mortality in highly immunosuppressed HIV patients.

Notably, we project that a CRAG screening program, including preemptive treatment, costs approximately $10.76 per person per year in Uganda. In comparison, the mean annual price for first line ART is $100 per year in sub-Saharan Africa, and delivery of ART including lab monitoring and personnel costs is estimated between $150 and $500 US dollars per person-year, suggesting that CRAG screening is relatively inexpensive and could serve as a key peri-ART intervention for immunosuppressed patients [49, 50].

## Conclusion

Taken together, our analysis suggests that CRAG screening would be a highly cost-effective policy intervention in Uganda, with potential application to much of sub-Saharan Africa. It also underscores the need for additional research such as case detection, rates of CM relapse and loss to follow-up in these regions. Further work should also focus on potential barriers to implementation and developing an optimized clinical algorithm for managing these patients. As access to ART continues to improve in these regions over time, median CD4 will rise and prevalence of CM will likely decrease – developing cost-effectiveness projections to model this progression will be useful in guiding allocation of care. As we move towards universal ART access, implementing CRAG screening has the potential to reduce the high burden of cryptococcal disease in Sub-Saharan Africa.

## Abbreviations

PLWH: People living with HIV
ART: Antiretroviral therapy
CM: Cryptococcal meningitis
CRAG: Cryptococcal antigen
DALY: Disability-adjusted life year
ICER: Incremental cost-effectiveness ratio
LFA: Lateral-flow assay
LP: Lumbar puncture
WTP: Willingness-to-pay
